# As-yet-uncultivated oral bacteria: breadth and association with oral and extra-oral diseases

**DOI:** 10.3402/jom.v5i0.21077

**Published:** 2013-05-23

**Authors:** José F. Siqueira, Isabela N. Rôças

**Affiliations:** Department of Endodontics and Molecular Microbiology Laboratory, Estácio de Sá University, Rio de Janeiro, Brazil

**Keywords:** uncultivated bacteria, oral microbiology, molecular biology methods, taxonomy

## Abstract

It has been shown that 40–60% of the bacteria found in different healthy and diseased oral sites still remain to be grown *in vitro*, phenotypically characterized, and formally named as species. The possibility exists that these as-yet-uncultivated bacteria play important ecological roles in oral bacterial communities and may participate in the pathogenesis of several oral infectious diseases. There is also a potential for these as-yet-uncultivated oral bacteria to take part in extra-oral infections. For a comprehensive characterization of physiological and pathogenic properties as well as antimicrobial susceptibility of individual bacterial species, strains need to be grown in pure culture. Advances in culturing techniques have allowed the cultivation of several oral bacterial taxa only previously known by a 16S rRNA gene sequence signature, and novel species have been proposed. There is a growing need for developing improved methods to cultivate and characterize the as-yet-uncultivated portion of the oral microbiome so as to unravel its role in health and disease.

It has been long recognized that >99% of the bacteria living on Earth cannot be successfully tamed in the laboratory (1–3). The issue of bacterial uncultivability has also been referred to as the ‘great plate count anomaly’, based on the observation that microscopic counts of bacteria in most environments are usually higher than the equivalent counts in culture (4). This can be regarded as one of the major challenges in contemporary microbiology, since if one cannot cultivate a certain microorganism, no definite information on its physiology and pathogenicity can be ascertained. Culture-independent molecular microbiology methods, especially those based on 16S rRNA gene clone library analysis, have been introduced in environmental microbiology to successfully identify virtually all bacterial members of communities established in diverse environments, including the as-yet-uncultivated portion (1, 5). Based on these studies, it has been estimated that 31 of the 61 distinct extant bacterial phyla still have no cultivable representatives (6).

Culture has been extensively used to analyze the bacterial communities associated with human body sites in health and disease. Even so, the introduction of molecular microbiology methods in the study of the human microbiome has revealed a taxonomic richness that is much larger than previously anticipated, with thousands of species per individual host. The importance of knowing the major microbial species that compose the human microbiome has been emphasized by the NIH Human Microbiome Project (7, 8), with the premise that this knowledge is essential for a full understanding of human healthy and diseased conditions.

Culture-independent molecular microbiology approaches have been widely used to scrutinize the human microbiome, with the significant advantage over culture-dependent methods of being able to identify as-yet-uncultivated microorganisms based on DNA signature sequences. Molecular studies have demonstrated that 20–80% of the species-level bacteria identified by 16S rRNA gene sequencing, depending on the site, still remain to be cultivated *in vitro* (9). For instance, surveys of the human gut microbiome have shown that each individual may harbor 500–3,000 bacterial species in the gut, with a high interindividual variation (10, 11). Eighty percent of the species composing the gut microbiome have not yet been cultivated and characterized (10, 12). As for other body sites, uncultivated phylotypes have been shown to represent about 40–50% of the species-level taxa detected in the esophagus (13), stomach (14), vagina (15, 16), and skin (17), although for the latter these figures may be less pronounced (18) (Table 1). The proportion of as-yet-uncultivated bacteria in the oral cavity is similarly high and is discussed in the next sections. All of these studies indicate that hundreds of species living in or on human sites are presently uncultivated. For virtually all of them, the only basic information available is a 16S rRNA gene sequence, which for many taxa is sufficient to infer phylogeny, but offers no information about morphology, metabolism, virulence, and so on.

**Table 1:** As-yet-uncultivated/uncharacterized phylotypes in different human body healthy and diseased sites. Data refer to the percentage of the total number of species-level taxa detected

Site	As-yet-uncultivated/uncharacterized bacteria,%	Study
Human oral cavity (diverse sites)	68	Dewhirst et al., 2010 (37)
Human oral cavity (nine different healthy sites)	60	Aas et al., 2005 (48)
Caries	33	Munson et al., 2004 (54)
	50	Aas et al., 2008 (67)
	10	Gross et al., 2012 (68)
Root caries	54	Preza et al., 2008 (66)
Subgingival plaque/periodontal disease	52	Kroes et al., 1999 (80)
	40	Paster et al., 2001 (47)
	60	Kumar et al., 2005 (79)
	59	de Lillo et al., 2006 (78)
	42	Aas et al., 2007 (81)
	19	Griffen et al., 2012 (90)
Peri-implantitis/periodontitis	69	Koyanagi et al., 2013 (88)
Peri-implantitis	46	Koyanagi et al., 2010 (110)
Tongue dorsum – halitosis	60	Kazor et al., 2003 (107)
Root canal infection (primary)	40	Munson et al., 2002 (55)
	67	Saito et al., 2006 (98)
	55	Sakamoto et al., 2006 (93)
	66	Ribeiro et al., 2011 (94)
Root canal infection (persistent/secondary)	55	Sakamoto et al., 2008 (148)
Extraradicular infection (endodontic origin)	36	Handal et al., 2009 (149)
Acute dental abscess	46	Sakamoto et al., 2006 (93)
	24	Flynn et al., 2006 (95)
	48	Riggio et al., 2007 (96)
Noma lesions	37	Paster et al., 2002 (150)
Other body sites Esophagus	38	Pei et al., 2004 (13)
Stomach	50	Bik et al., 2006 (14)
Vagina	47	Verhelst et al., 2004 (15)
	45	Fredricks et al., 2005 (16)
Skin	50	Dekio et al., 2005 (17)
Gut	76	Suau et al., 1999 (12)
	80	Eckburg et al., 2005 (10)

In addition to composing large portions of the microbiome in health, as-yet-uncultivated bacteria have also been detected in association with diseased sites and a role in pathogenesis has been suspected. Examples of diseased conditions where uncultivated bacterial phylotypes have been found include chronic wounds (19, 20), vaginosis (21), aortic aneurysms (22), corneal ulcer (23), bone and joint infections (24), cystic fibrosis (25), sinusitis (26), and intra-amniotic inflammation leading to preterm birth (27). Bacteria with no cultivated representatives have been found in health and virtually every infectious disease in the oral cavity, and are discussed later in this article.

## As-yet-uncultivated bacterial phylotypes

For the purpose of this review, we define bacterial species that have not yet been grown in the laboratory as as-yet-uncultivated bacteria. The terms ‘uncultivable’ or ‘non-culturable’ are avoided since many species have not been cultivated merely by chance and because conceptually all bacteria can grow under proper nutritional and physicochemical conditions (28). The term ‘phylotype’ is used throughout this article to refer to those as-yet-uncultivated species that are known only by a 16S rRNA gene sequence.

Bacteria that are difficult or so far impossible to cultivate can be classified into two non-exclusive categories: the as-yet-uncultivated phylotypes and the viable but not cultivable (VBNC) bacteria (29). The former category consists of bacterial species with no cultivated representatives, which are known only by a 16S rRNA gene sequence and for which adequate conditions for culture have still to be determined. They are the subject of this review. Bacteria that have already been cultivated and characterized, but whose cells may enter a dormant non-dividing state when exposed to certain conditions represent the latter category. These cells, while still alive, do not form visible colonies on the surface of solid media nor increase the turbidity of culture broth (30). Molecular methods for specific detection of certain cultivable oral species have demonstrated higher prevalences than culture (31–36). In addition to other reasons, such as the higher sensitivity (detecting fewer cells in a sample) of molecular methods when compared to culture and not discarding the possibility of detection of DNA from dead cells in certain conditions, the presence of cells in a VBNC state may help explain the higher prevalence of cultivable species as determined by molecular methods.

As-yet-uncultivated phylotypes cannot be given a formal species name, since naming a species requires cultivation for phenotypic characterization. There is no rule for naming phylotypes, so there may be a huge redundancy when evaluating separate studies, with different authors giving different names for the same phylotypes. Redundancy becomes quite evident when one searches large public databases, such as the GenBank, for identification of an unknown 16S rRNA gene sequence retrieved from a clinical sample. In some cases, tens of hits with 100% sequence similarity are returned. In order to organize this information for oral bacteria, Dewhirst et al. (37) created a provisional naming system termed the human oral microbiome database (HOMD). This system allows for comparisons between different studies and adequate communication between researchers, helping to strengthen the association of certain phylotypes with diseased conditions.

It is very important to expend efforts towards the cultivation of many of these phylotypes. By culturing these bacteria, one may obtain information about their functional and ecological role in communities associated with health or disease, as well as the susceptibility patterns to antibiotics and other antimicrobial agents. Methods that have been developed to cultivate as-yet-uncultivated bacteria are discussed later in this article. As a result of these efforts, there has been a substantial increase in the number of cultivated species over the last 10 years (29).

## Diversity of the oral microbiome

The oral microbiome is composed of diverse microbial groups, including bacteria, fungi, archaea, viruses, and protozoa (38). Bacteria are the dominant microorganisms and consequently have been subject of extensive investigation. Comprehensive culture analyses of different oral sites have disclosed a high bacterial diversity (39). Approximately, 280 of the oral bacterial species have been isolated in culture and validly named (40). However, limitations of culture in revealing the actual diversity of the oral microbiome have been apparent for over a century. A study published in 1894 by Willoughby Dayton Miller (41) reported the occurrence of uncultivated bacteria in association with root canal infections. Most of the bacteria that Miller had observed using light microscopy could not be grown *in vitro* using the technology available at that time. He wrote: ‘many species of bacteria occurring in the diseased pulp, vibriones, spirochaetes, the stiff pointed bacilli and threads, have not been found cultivable on artificial media anyway; and possibly there are still other uncultivable pulp-bacteria’ (41). Later, a study by Socransky et al. (42) using light microscopy and culture suggested that roughly one-half of the oral microbiome could not be cultivated in the laboratory. Introduction of culture-independent nucleic acid methods to the analysis of oral bacterial diversity has not only confirmed this picture revealed by microscopic studies, but also demonstrated a still broader and more diverse spectrum of extant oral bacteria.

According to the HOMD, the human oral microbiome comprises 619 species-level prokaryotic taxa belonging to 13 phyla, namely *Actinobacteria*, *Bacteroidetes*, *Chlamydiae*, *Chloroflexi*, *Euryarchaeota*, *Firmicutes*, *Fusobacteria*, *Proteobacteria*, *Spirochaetes*, SR1, *Synergistetes*, *Tenericutes*, and TM7 (37). The largest majority of species-level taxa in the oral cavity (about 96%) fall into the phyla *Firmicutes*, *Bacteroidetes*, *Proteobacteria*, *Actinobacteria*, *Spirochaetes*, and *Fusobacteria* (37). Estimates indicate that the overall number of oral species may be even higher, reaching approximately 1,200 species (43). Moreover, studies using next-generation sequencing technologies have reported representatives belonging to approximately twice as many as the number of phyla listed in HOMD (44, 45). Because the resolution of these methods has not allowed accurate identification of sequences to the species level and considering that data from pyrosequencing analyses may overestimate diversity (46), this review article will focus mostly on the taxonomic data available in HOMD.

Studies based on 16S rRNA gene clone libraries have shown that 40–60% of the oral microbiome is composed of as-yet-uncultivated bacteria (37, 47, 48) (Table 1). HOMD lists approximately 220 oral taxa that have not been cultivated so far (49). It has been shown that only 29–50% of the oral species-level taxa belonging to the phyla *Firmicutes*, *Proteobacteria*, *Bacteroidetes*, *Actinobacteria*, and *Fusobacteria* have been successfully cultivated. The number of cultivated members of the phyla *Spirochaetes* and *Synergistetes* is still very low (37, 50). Of 48 oral treponemes listed in HOMD, only 10 have been cultivated and named. Of the 10 oral *Synergistetes* in HOMD, only three have been recently cultivated and validly named: *Jonquetella anthropi* (51), *Fretibacterium fastidiosum*, (52) and *Pyramidobacter piscolens* (53). Moreover, the phyla TM7, SR1, and *Chloroflexi* have no cultivated oral representative (37). All these findings demonstrated that several bacterial species inhabiting the oral cavity may have been overshadowed by limitations of culture techniques. This raises the interesting possibility that as-yet-uncultivated and unnamed species may actually play an important ecological, beneficial, or pathogenic role in the oral cavity. Thus far, their role in health and disease can only be inferred on the basis of association data from studies using molecular microbiology methods.

## Reasons for ‘uncultivability’

Many bacteria may have not been cultivated and phenotypically characterized merely by chance. For instance, a given species may occur in low abundance in the environment and pass unnoticed as more dominant species are identified. Also, slow-growing species may be overcome by fast-growing ones and remain undetected. Another reason is that some species may be difficult to identify by phenotype-based approaches and may have been identified in previous studies only to the genus level or even misclassified. Studies using culture followed by identification of the isolates by 16S rRNA gene sequencing have revealed many species-level taxa that were previously identified only by culture-independent approaches and regarded as uncultivated phylotypes (54–57). For instance, our group used ordinary anaerobic culture coupled with 16S rRNA gene identification for analysis of endodontic infections and found isolates from the genera *Prevotella, Fusobacterium*, and *Actinomyces* that were previously deemed as uncultivated phylotypes (56). In a comprehensive study of the bacteria isolated from severe early childhood caries, Tanner et al. (57) identified more than 5,000 isolates using 16S rRNA gene sequencing and found 45 previously uncultivated taxa, 29 extended HOMD taxa, and 45 potential novel groups; most of the previously uncultivated taxa belonged to the genera *Streptococcus*, *Selenomonas*, *Actinomyces*, and *Capnocytophaga*.

Although these studies suggest that many species may have been overlooked by culture methods by coincidence, one must realize that the problem of ‘uncultivability’ is real. It is not difficult to understand that many bacteria cannot thrive in the unfamiliar and artificial conditions of *in vitro* culture (58). Most bacteria live in their environments in biofilm communities, which are characterized by a network of interbacterial communications, with a multitude of interactions among the community members themselves and between them and the environment (or host). In the natural environment, optimal conditions for growth are met, including nutrients, growth factors, signaling molecules, oxygen tension, and other physicochemical conditions. For the same bacteria to be cultivated in the laboratory, there is a need for these conditions to be properly reproduced.

There are several possible reasons for the fact that many bacterial species remain to be grown *in vitro* and phenotypically characterized. They include (6, 59–62):

1. lack of essential nutrients, growth factors, and/or signaling molecules in the artificial culture medium;
2. overfeeding conditions. Most culture media used for open-ended bacterial cultivation are nutrient-rich. Under such conditions, slow-growing species may be overcome by faster-growing species. In addition, some species are highly adapted to environments with low availability of nutrients and excess of nutrients may also generate excess of metabolic end-products, which may reach toxic levels to the cells;
3. toxicity of the culture medium itself, which can inhibit the growth of some species;
4. metabolic dependence on other species for growth, which may be established by cross-feeding or metabolic cooperation to degrade complex substrates;
5. disruption of bacterial quorum-sensing and other signaling systems induced by separation of bacteria from biofilm communities on solid culture media.

## As-yet-uncultivated bacteria in oral healthy conditions

A large number of species found in the healthy oral cavity still remains to be cultivated and phenotypically characterized. In a comprehensive study using 16S rRNA gene amplification, cloning, and sequencing, Aas et al. (48) evaluated the breadth of bacterial diversity in nine sites from clinically healthy subjects. They reported that as-yet-uncultivated and unnamed species-level taxa corresponded to approximately 50% of the species in samples from the tongue dorsum and lateral sides of tongue, 30% from the buccal epithelium, 50% from the hard palate, 40% from the soft palate, 50% from the supragingival biofilm, 35% from the subgingival biofilm, 30% from the maxillary anterior vestibule, and 40% from tonsils. Overall, they found 141 predominant species in oral healthy sites, of which over 60% have not been cultivated.

## As-yet-uncultivated bacteria and oral disease

Most endogenous infections in the human body are caused by multispecies biofilms composed of bacteria that are usually normal inhabitants of the human body surfaces and cavities (63). These include the most common oral infections: caries, periodontal diseases, and apical periodontitis. Bacterial phylotypes that still remain to be cultivated and characterized have been found in association with these diseases and virtually all the other oral infectious diseases caused by bacteria. Table 2 provides examples of several as-yet-uncultivated bacterial phylotypes that have been found in different oral diseased sites.

**Table 2:** Examples of as-yet-uncultivated/uncharacterized oral bacteria found in association with diseased sites

As-yet-uncultivated/uncharacterized phylotypes (HOMD classification)	Synonym	GenBank accession no.	Oral condition	Study
*Actinobacteria*				
*Actinobaculum* sp. HOT-183^1^	*Actinobaculum* oral clone EL030	AY008311	Root caries	(66, 100)
			Root canal	
*Actinomyces* sp. HOT-169^1^	*Actinomyces* oral clone AG004	AF287747	Caries	(69)
*Actinomyces* sp. HOT-170^1^ ^2^	*Actinomyces* oral clone AP064	AF287749	Caries	(69, 151)
			Root canal	
*Actinomyces* sp. HOT-175^1^	*Actinomyces* oral clone GU067	GU407261	Caries	(67)
*Actinomyces* sp. HOT-448^2^	*Actinomyces* oral clone IP073	AY349365	Root caries	(66)
*Atopobium* sp. HOT-199^1^	*Atopobium* oral clone C019	AF287760	Root canal	(152)
*Atopobium* sp. HOT-416^1^ ^2^	*Atopobium* genomospecies C1	AY278623	Caries	(67, 73, 93, 94, 148)
			Root canal	
*Bacteroidetes*				
*Bacteroidaceae* sp. HOT-272^1^	*Bacteroidetes* oral clone X083	AY005066	Root canal Extraradicular infection Periodontal disease	(47, 93, 94, 98, 104, 148, 149, 153, 154)
*Bacteroidales* sp. HOT-274^1^ ^2^	*Bacteroidetes* oral clone AU126	AY005072	Periodontal disease Root canal Extraradicular infection	(82, 86, 148, 149, 155)
*Bacteroidetes* sp. HOT-365^1^	*Bacteroidales* oral clone E2b MCE7_164	AF481206	Root canal	(55, 93, 98, 154)
*Prevotella* sp. HOT-304^1^	*Prevotella* oral clone DA058	AY005065	Root canal Peri-implantitis	(88, 156)
*Prevotella* sp. HOT-306^1^	*Prevotella* oral clone DG059	GU409581	Caries Root canal	(69, 157)
*Prevotella* sp. HOT-472^1^	*Prevotella* oral clone GU027	GU413272	Root canal	(56, 152)
*Firmicutes*				
*Erysipelotrichaceae* sp. HOT-904^1^	*Solobacterium* oral clone 6Ta-2	AB256031	Root canal	(94, 152)
*Lachnospiraceae* sp. HOT-086^1^	*Eubacterium* oral clone BU061	AF385567	Root canal	(98, 156)
*Lachnospiraceae* oral clone 55A-34 (no HOMD)^1^	*Lachnospiraceae* oral clone 55A-34	AB213385	Root canal Peri-implantitis	(88, 93, 105, 110)
*Megasphaera* sp. HOT-123^1^ ^2^	*Megasphaera* oral clone CS025	AF287784	Root canal Periodontal disease	(91, 93)
*Mitsuokella* sp. HOT-131^1^ ^2^	*Selenomonas* oral clone CS002	AF287795	Root caries Root canal	(66, 156)
*Moryella* sp. HOT-373^1^	*Lachnospiraceae* oral clone E4 MCE9_173	AF481221	Root canal	(55, 98, 152, 154)
*Oribacterium* sp. HOT-102^1^	*Lachnospiraceae* oral clone E1 MCE7_60	AF481218	Root canal	(55, 93, 94, 98)
*Peptostreptococcaceae* sp. HOT-103^1^ ^2^	*Eubacterium* oral clone PUS9.170	AJ012604	Periodontal disease Root canal	(47, 98, 158, 159)
*Peptostreptococcaceae* sp. HOT-383^1^	*Eubacteriaceae* oral clone P3 P2PB_46	AF538856	Root canal	(98, 148, 154, 156)
*Selenomonas* sp. HOT-126^1^ ^2^	*Selenomonas* oral clone EY047	AF385576	Caries	(67)
*Selenomonas* sp. HOT-146^2^	*Selenomonas* oral clone EW084	AF385503	Periodontal disease	(83, 85)
*Selenomonas* sp. HOT-149^1^	*Selenomonas* oral clone FT050	AY349403	Periodontal disease Root caries Root canal	(66, 83, 156)
*Streptococcus* sp. HOT-058^1^ ^2^	*Streptococcus* oral clone BW009	AY005042	Caries Tongue dorsum – halitosis Extraradicular infection	(107, 149, 160)
*Streptococcus* sp. HOT-064^1^	*Streptococcus* genomosp. C8	AF385574	Caries	(160)
*Streptococcus* sp. HOT-071^1^	*Streptococcus* genomosp. C3	AY278631	Caries	(160)
*Veillonella* sp. HOT-780^2^	*Veillonella* oral clone HB016	DQ087189	Caries	(160)
*Veillonellaceae* sp. HOT-132^1^	*Selenomonas* oral clone CS015	AF287791	Root canal Peri-implantitis	(88, 97)
*Veillonellaceae* sp. HOT-155^2^	*Selenomonas* oral clone GAA14	AF287789	Periodontal disease Root caries	(47, 66)
*Proteobacteria*				
*Desulfobulbus* sp. HOT-041^1^ ^2^	*Desulfobulbus* oral clone R004/CH031	AY005037/AY005036	Periodontal disease Root canal Peri-implantitis	(47, 79, 84, 90, 94, 98–100, 110, 151)
*Spirochaetes*				
*Treponema* sp. HOT-231^1^ ^2^	*Treponema* sp. I:G:T21	AF023055	Periodontal disease Root canal	(47, 105)
*Treponema* sp. HOT-258^1^	*Treponema* sp. IV:18:C9	AF023042	Root canal	(103)
*Treponema* sp. HOT-269^1^ ^2^	*Treponema* sp. 6:H:D15A-4	AY005083	Periodontal disease Root canal	(79, 98, 161)
*Synergistetes*				
*Fretibacterium* sp. HOT-359^1^ ^2^	*Synergistes* oral clone BH007	AY005447	Periodontal disease Extraradicular infection	(79, 149)
*Fretibacterium* sp. HOT-360/HOT-362^1^ ^2^	*Synergistes* oral clone BH017/D084	AF125199/AF125200	Periodontal disease Root canal Extraradicular infection	(47, 82, 86, 90, 98–100, 149)
TM7				
TM7 sp. HOT-352^2^	TM7 oral clone DR034	AF385520	Tongue dorsum – halitosis	(107)
TM7 sp. HOT-349^2^	TM7 oral clone BS003	AY005448	Periodontal disease	(90, 91)
TM7 sp. HOT-356^1^ ^2^	TM7 oral clone I025	AF125206	Periodontal disease Root canal Peri-implantitis	(47, 82, 87, 88, 99, 100, 123)
SR1				
SR1 sp. HOT-345^2^	SR1 oral clone X112	AF125207	Periodontal disease	(82, 155)

1 Detected in the diseased site.

2 Higher prevalence/abundance in disease than health.

### Caries

The etiopathogenesis of different forms and stages of caries have been clearly associated with cultivable species of *Streptococcus*, *Lactobacillus*, and *Actinomyces* (64, 65). Nonetheless, molecular microbiology studies have shown a microbiome that is much more complex than previously reported by culture studies, and several other species/phylotypes have been included in the list of potential caries pathogens. About 33–54% of the species-level taxa detected in caries lesions have not been cultivated so far (54, 66, 67), while a more recent study reported 10% of uncultivated taxa (68). As-yet-uncultivated phylotypes or uncharacterized strains of *Bifidobacterium*, *Propionibacterium*, and *Atopobium* have been associated with caries and regarded as potential pathogens (66, 67, 69). Advanced dentinal caries lesions have been shown to be dominated by lactobacilli and/or species/phylotypes of the genera *Prevotella*, *Selenomonas*, *Dialister*, *Fusobacterium*, *Eubacterium*, *Olsenella*, *Atopobium*, *Bifidobacterium*, as well as members of the *Lachnospiraceae* family (70–73).

### Periodontal diseases

Periodontal diseases result from the subgingival presence of complex bacterial biofilms, and some cultivable species have been considered as the major periodontal pathogens based on both culture and molecular studies: *Porphyromonas gingivalis, Tannerella forsythia, Treponema denticola*, and *Aggregatibacter actinomycetemcomitans* (74–77). Molecular studies have included several new species in the list of candidate periodontal pathogens. These studies revealed that 40–60% of the periodontal microbiome are made up of as-yet-uncultivated species-level phylotypes (47, 78–81). Examples of uncultivated bacteria found in association with periodontal diseases include phylotypes of the genera *Prevotella*, *Selenomonas, Desulfobulbus*, *Peptostreptococcus, Treponema, Fusobacterium*, as well as members of the *Lachnospiraceae* family and the phyla *Bacteroidetes*, *Synergistetes*, TM7, and SR1 (47, 79, 82–91).

### Endodontic infections

Similar to caries and periodontal diseases, the breadth of bacterial diversity in endodontic infections has been substantially expanded by culture-independent molecular methods (92). Clone library analyses of different types of endodontic infections reveal that a significant proportion of the microbiome consists of not-yet-cultivated bacteria (55, 93, 94). Sakamoto et al. (93) reported that uncultivated phylotypes accounted for approximately 55% of the taxa found in root canals of teeth with apical periodontitis and in terms of abundance represented more than 38% of the clones sequenced. In pus aspirates from acute apical abscesses, as-yet-uncultivated phylotypes encompassed approximately 24–46% of the taxa found (93, 95), and 6% to >30% of the clones sequenced (93, 96). Uncultivated phylotypes from several genera have been identified, including *Dialister*, *Treponema, Prevotella*, *Solobacterium*, *Olsenella*, *Fusobacterium*, *Eubacterium*, *Megasphaera*, *Veillonella*, and *Selenomonas* as well as phylotypes related to the family *Lachnospiraceae* or the *Synergistetes* and TM7 phyla (55, 93) (97–103). One of the most prevalent as-yet-uncultivated phylotypes found in endodontic infections is *Bacteroidaceae* sp. HOT-272 (synonym, *Bacteroidetes* oral clone X083) (104, 105).

### Other oral conditions

Colonization of the tongue dorsum by bacteria that produce volatile sulfur compounds and other metabolites represents a major source of oral malodor in individuals with halitosis (106). Molecular studies evaluating the microbiome associated with halitosis have shown a large proportion of as-yet-uncultivated taxa, including phylotypes of the genera *Dialister* and *Streptococcus*, and the phylum TM7 (107, 108). Tonsilloliths, which are another potential cause of halitosis, can be colonized by a microbiome composed mostly of anaerobic bacteria that produce volatile sulfur compounds as well as several uncultivated phylotypes (about 50% of the species-level taxa detected) (109).

Other examples of oral diseased conditions that present a potential involvement of uncultivated bacteria include peri-implantitis and osteoradionecrosis. The microbiome of peri-implantitis has been shown to be more diverse than that of periodontitis and about one-half of the species found were uncultivated phylotypes (88, 110). As for osteoradionecrosis, a study evaluating the bacteria associated with necrotic bone lesions of the mandible after radiation therapy reported that 27% of the species-level taxa detected have not been cultivated (111).

### Uncultivated oral bacteria in extra-oral infections

Oral bacterial species have been detected in distant body sites and suggested to be related to a variety of systemic diseases (112–114). It is entirely possible that it is also true for as-yet-uncultivated oral phylotypes. For instance, uncultivated oral phylotypes have been detected in blood samples in episodes of bacteremia following dental procedures (115). They included representatives of the genera *Streptococcus*, *Actinomyces, Veillonella*, and *Prevotella*. Uncultivated oral phylotypes have been detected in samples from non-oral diseased sites, as for instance in brain abscess (116), ventilator-associated pneumonia (117), sinusitis (26), sputa from cystic fibrosis patients (25), and intrauterine infection leading to preterm birth (118). The role of these species that still remain to be cultivated and characterized in extra-oral diseases is another challenging aspect of the study of oral bacteria.

## Cultivating the ‘uncultivable’

The history of microbiology has plenty of examples of bacteria that were, at a certain time, regarded as uncultivated but after further technological developments were successfully grown in the laboratory and characterized. In this regard, anaerobes are a very good example. By the end of the 19th century, microscopic studies revealed oral bacteria that could not be grown with the methods available at that time. Advances in techniques for culture of anaerobic bacteria unearthed a myriad of species, many of which demonstrated to be very important pathogens in several infectious diseases. Recent refinements in culturing and identification methods have allowed cultivation and characterization of some species that had only previously been detected and identified by a 16S rRNA gene sequence.

### Newly cultivated and characterized species

Until very recently, there are many examples of bacterial species that were considered as uncultivable and that have been further successfully cultivated, phenotypically characterized, and named. These can be either bacteria that are relatively easy to cultivate on ordinary media but have, for whatever reason, only recently been cultivated for the first time (e.g. *Prevotella baroniae, Peptostreptococcus stomatis, Dialister invisus, Anaeroglobus geminatus*, etc.) or bacteria that are truly resistant to culture (e.g. *Fretibacterium fastidiosum*). Strategies to cultivate the latter are discussed in the next section.

Many of these newly named species have been associated with disease. *Scardovia wiggsiae*, which was formerly referred to as *Bifidobacterium* clone CX010 or *Scardovia* genomospecies C1, has been found in association with severe early childhood caries and dentinal caries (54, 57, 119). *Dialister invisus*, which is synonymous with *Dialister* E1 strains E2.20, E3.07, E9.48, P2.65, E7.25, and clones GBA27, IS013B24, BS095, and 9N-1, has been frequently detected in infected root canals and abscesses, as well as in the subgingival biofilm in periodontal diseases (55, 91, 102, 120, 121). Three recently cultivated members of the *Synergistetes* phylum that have been associated with endodontic infections and periodontal diseases include *Piramydobacter piscolens* (formerly oral clone BA121, P4G_18 P1, genomosp. C1), *Fretibacterium fastidiosum* (oral clones W028 and W090), and *Jonquetella anthropi* (oral clone E3_33) (47, 79, 122, 123). *Prevotella baroniae* was previously identified by molecular methods as *Prevotella* clones PUS9.180 and E9_42-E4 and has been encountered in high prevalence in abscesses of endodontic origin (104, 124). Other examples include *Peptostreptococcus stomatis* (oral clone CK035), *Anaeroglobus geminatus* (*Megasphaera* oral clone BB166), and *Propionibacterium acidifaciens* (strain FMA5), which have been reported to occur in periodontal pockets, infected root canals, and caries lesions (66, 73, 79, 82, 105, 125).

### Strategies to cultivate as-yet-uncultivated bacteria

It must be assumed that no single method or culture medium is suitable for isolating the vast diversity of bacteria present in most environments (126). There has been a growing trend to develop specific approaches and culture media that allow cultivation of previously uncultivated bacteria.

Methods like the fluorescence in situ hybridization (FISH) approach and its derivations permit that uncultivated bacteria be directly visualized in clinical specimens (2, 127–129). By using oligonucleotide probes designed to target specific phylotypes, one can have information about the morphology of the cells, their spatial location in the tissues, as well as their physical relationship to the host tissues and other bacteria in a multispecies community. These approaches have demonstrated that uncultivated oral *Synergistetes* cells are large curved bacilli (127), uncultivated *Tannerella* phylotypes BU045 and BU063 are long slim rods with segments (129), and TM7 cells are long, thick filaments (87, 128). Nonetheless, due to the fact that processing of the specimens for these techniques involves steps that kill the cells, one cannot selectively isolate the uncultivated bacteria for further cultivation and characterization.

Strategies to cultivate the so-called uncultivated bacteria may rely on application of conditions that are as close as possible to the natural environment from which the samples were taken. Recent efforts to accomplish this have met with some success. Examples of strategies to culture the uncultivated portion of environmental communities include the following:

1. Use of culture media with little or no added nutrients (6, 130). This is because traditional culturing procedures are usually based on excessive nutrient supply, which results in the overgrowth of fast-growing and less fastidious species. Species fastly growing in enriched non-selective media may mask or inhibit the growth of less dominant and rarely cultivated or uncultivated bacteria (61).
2. Long-term cultivation (6, 29). It has been shown that strains from the SAR11 clade could be successfully cultivated after extended incubation from 8 to 24 weeks (131). Strains of the TM7 phylum have also been reported to be cultivated on low-nutrient solid media after a 50-day incubation (132).
3. Cultivation of slow-growing bacteria may be improved by serial dilution to extinction. This approach leads to reduction of the inoculum size and decreases the chances of competition by faster-growing species present in the community (29, 130).
4. Addition of specific growth factors in the culture media. Examples include hemin and menadione for optimal growth of many dark-pigmented oral bacterial species (133), pyridoxal or L-cysteine for *Abiotrophia* and *Granulicatella* species (134), and N-acetyl muramic acid for *Tannerella forsythia* (135). Some factors may be provided by growing the target species in co-culture with a feeder species. For growth of the newly cultivated and characterized *Fretibacterium fastidiosum*, there is a need for co-culture with, or extracts from, selected other oral species, such as *Fusobacterium nucleatum* (52). A great challenge for most uncultivated bacteria is to determine the specific substances present in the natural environment that are required for growth *in vitro*.
5. *In vivo* incubation. A very interesting strategy to ensure the availability of natural growth factors is to perform incubation in the natural environment using special devices (130, 136, 137). Examples of such devices include a diffusion chamber (137, 138) or a hollow fiber membrane chamber (139). These approaches permit the diffusion and distribution of growth factors from the natural environment into the culture media through a membrane. Another method consists of encapsulating single cells in gel microdroplets, which are exposed to the natural environment (140). After growth, gel microdroplets containing the bacterial colonies can be sorted by flow cytometry.

Recent studies have used some of these approaches or a combination thereof to cultivate some previously uncultivated oral bacteria. Vartoukian et al. (141) used the colony hybridization method to grow and isolate an uncultivated *Synergistetes* strain. This strain grew in Cooked Meat Medium in co-culture with other subgingival biofilm bacteria. The colony hybridization method was carried out using membrane transfers from plate cultures. Hybridization detections on membranes were used to locate matched microcolonies within mixed cultures, and the subcultured colonies were further grown with feeder species as inferred from the original mixed culture.

Sizova et al. (130) used several approaches to culture uncultivated oral bacteria, including in vivo cultivation by the ‘minitrap’ method, single-cell long-term cultivation, and modifications of conventional enrichment techniques with media containing no sugar. They reported that the most successful recovery was achieved using the ‘minitrap’ approach, followed by single-cell cultivation, and then conventional plating. In terms of species richness, the single-cell cultivation method showed superior results. The methods were complementary to each other, with no single species being isolated by all of them. Using these methods, the authors succeeded in isolating and maintaining in pure culture 10 strains known previously only by their 16S rRNA gene sequences. In addition, representatives of three novel genera that are new to the oral cavity were disclosed.

Some sophisticated strategies to devise specific culture media for as-yet-uncultivated bacteria have the potential to be widely available in the near future. Procedures have been developed that allow sequencing of the complete genome of individual bacterial cells, including uncultivated phylotypes, directly from their natural environment (142, 143). Isolation of individual cells have been successfully achieved by fluorescence-activated cell sorting, micromanipulation, microfluidic devices, or serial dilution, followed by whole genomic amplification using techniques such as multiple-strand displacement amplification (142, 144, 145). The amplified genome is then sequenced. The availability of sequenced genomes of as-yet-uncultivated bacteria provides opportunities to define culture media for growth of these bacteria with computer modeling of metabolic networks. For instance, by analyzing the sequenced genome of bacteria recalcitrant to culture, one can identify missing genes and consequent metabolic deficiencies and use this information to develop a culture medium containing substances that complement such deficient metabolic pathways (146).

### Concluding remarks

A large proportion of species found in the oral cavity in healthy and diseased conditions still remain to be grown *in vitro*, phenotypically characterized, and then formally named as a species. The fact that these bacteria have not yet been cultivated and characterized does not mean that they are not important. It is reasonable to surmise that species that play an important ecological role in mixed communities can be overlooked if they cannot be cultivated. Moreover, several of the as-yet-uncultivated oral phylotypes have been regarded as suspected pathogens involved in diverse oral infections based on association data. This suggests that they can be previously unrecognized bacteria that play a role in the pathogenesis of different oral infectious diseases. However, association does not necessarily translate into a cause-and-effect relationship, since the possibility also exists that these bacteria may be secondary colonizers that took advantage of the environment changed by disease. For a comprehensive characterization of physiological and pathogenic properties of individual bacterial species, as well as determination of susceptibility to antimicrobial agents, there is a need for growing strains in pure culture. Advances in culturing techniques have allowed the cultivation of several oral bacteria only previously known by a 16S rRNA gene sequence signature, and novel species have been proposed. In addition, given the large proportion and diversity of uncultivated bacteria, the possibility that several of these bacteria may be source of potential novel drugs is ready to be explored by new and emerging technologies (147).
